# The use of the free vascularised bone graft for nonunion of the scaphoid: a systematic review

**DOI:** 10.1186/1749-799X-9-21

**Published:** 2014-04-01

**Authors:** Talal Al-Jabri, Ashim Mannan, Peter Giannoudis

**Affiliations:** 1 Department of Surgery and Cancer, Imperial College London, London SW7 2AZ, UK; 2 Department of Trauma and Orthopaedics, Leeds General Infirmary, Great George St, Leeds LS1 3EX, UK; 3 Department of Trauma and Orthopaedics, Diana Princess of Wales Hospital, Grimsby DN33 2BA, UK

**Keywords:** Scaphoid, Nonunion, Graft, Vascularised, Free

## Abstract

**Background:**

Fractures of the scaphoid are well known to be problematic especially when complicated by avascular necrosis, nonunion and carpal collapse. Fixation techniques have involved nonvascularised bone grafting; however, in the presence of avascular necrosis, generally poor union rates (47%) occur as identified by a meta-analysis performed by Merrell et al. The introduction of pedicled vascularised bone grafts showed further improvement; however, in the presence of carpal collapse, union rates as low as 50% have been reported by Chang et al. amongst others using the 1,2-intercompartmental supraretinacular artery pedicled graft. The difficulty lies in having a short pedicle with limited manoeuvrability to correct a humpback deformity and insert into the scaphoid cavity. Prior trauma to the soft tissues or distal radius may prohibit the use of pedicled grafts. The aim of this systematic review is to examine the published evidence for the use of free vascularised bone grafts in cases of scaphoid nonunion.

**Methods:**

A systematic review was performed with the following defined search strategy on MEDLINE and Google Scholar: ((scaphoid nonunion) OR scaphoid pseudarthrosis) AND bone graft. Articles were reviewed and data compiled into tables for analysis. Statistical analysis was performed with determination of descriptive statistics, and differences between the groups were calculated using categorical variables and chi-square test. A *p* value of 0.05 or less was considered to be statistically significant.

**Results:**

Two hundred and sixty-three articles were identified with a total of 12 articles meeting the inclusion criteria. Two hundred and forty-five cases of scaphoid nonunion were identified through the articles included in this systematic review. Fifty-six patients underwent free vascularised bone grafts from the medial femoral condyle with a 100% union rate and correction of humpback deformity, and 188 patients underwent free vascularised bone grafting from the iliac crest with an 87.7% union rate. The difference between the two similar groups was statistically significant (*p* = 0.006).

**Conclusions:**

The promising data suggests that the medial femoral condylar free graft based on the descending genicular vessels can be considered in cases of proximal pole avascular necrosis and humpback deformity or in situations where other flaps are precluded or deemed unlikely to cause union.

## Introduction

The scaphoid is the most commonly fractured carpal bone accounting for 60% of fractures [1,2]. Whilst most scaphoid fractures may heal, a number of case series have identified a 10%–15% nonunion rate [1]. Nonunion is directly related to an interruption of the tenuous blood supply to the scaphoid; therefore, the major risk factor for nonunion is fracture displacement which has been associated with a nonunion risk of up to 55% in patients [2]. Other major risk factors include proximal fracture location, soft tissue interposition, inadequate immobilisation and the presence of avascular necrosis (AVN) [2,3].

Subsequent to nonunions, progressively degenerative changes may occur with the formation of cysts, bony resorption with loss of bone stock and the development of apex dorsal angulation or the humpback deformity [4]. Ultimately, this may lead to scaphoid nonunion advanced collapse (SNAC) of the wrist and the formation of a proximal pole which extends with the lunate. The resultant wrist architecture is known as a dorsal intercalated segment instability (DISI) deformity. This has serious functional implications for the patient in terms of wrist range of movement, grip strength and general activities of daily living [4,5].

The management of nonunion has remained controversial since the last century with the likes of Cole and Williamson emphatically stating:

Bone grafting of the fragments has been done with some success but will never be a choice for routine procedure and it is mainly a surgical stunt [6]*.*

However, opinion gradually changed when methods introduced by Russe and others showed promising results in the early 1950s [7]. Indeed, bone grafting has been performed since the late 1920s with positive results [7]. Though, one must classify the different types of bone grafting used. This treatment modality may include nonvascularised bone grafts (NVBGs) which may be cortical or cancellous and autograft or allograft. With the introduction of NVBGs, a major improvement in union rates was heralded when combined with internal fixation [8]. This was a major stepping stone in the surgery for scaphoid nonunions; however, there were some major limitations, and there was a wide range in terms of union rates. The importance of vascularity was enforced by finding that in the presence of AVN, conventional NVBGs could only achieve a 47% union rate [9]. However, in the absence of AVN, these NVBGs could achieve union rates of 94% [9].

There was growing consensus that new techniques were required to address the shortfall, and accordingly, vascularised bone grafting (VBG) techniques stemmed from this. It was widely believed that providing adequate blood flow would help treat cases of nonunion. Several *in vivo* studies were able to demonstrate that VBGs accelerated bone healing by preserving osteocytes and preventing the slower creeping substitution, and (as was hoped) canine models were able to demonstrate increased blood flow and superior mechanical properties in VBGs as opposed to NVBGs [10-19]. Studies have also shown that vascularised periosteal grafts have osteogenic capacity and form new bone, and inclusion of cortical bone or overlying muscle further improves graft viability [11,18,19].

VBGs could be further classified into pedicled or free VBGs. Pedicled VBGs involve isolating a segment of bone local to the defect and maintaining the blood supply to this segment of donor bone which is then fixed into the recipient site. This requires a good stock of donor bone in close proximity to the defect.

However, although dorsal and volar pedicled VBGs have enjoyed some success, they are the victims of their own limitations. Soon after their introduction, it became apparent that in cases of carpal collapse or humpback deformity, the commonly used dorsal distal radius pedicled VBGs are limited. Chang et al. was able to show only a 50% success rate for the widely used VBGs based on the 1,2-intercompartmental supraretinacular artery (1,2-ICSRA) in the presence of carpal collapse [20].

The difficulty lies in having a short pedicle and a limited manoeuvrability of the graft to correct a humpback deformity and insert into the scaphoid cavity. Additionally, prior trauma to the soft tissues or distal radius may prohibit the use of local VBGs. The vascularity of the common pedicled VBGs has also been noted to be unreliable [21]. The use of free VBGs may overcome these challenges. Free VBGs involve detaching a segment of bone with its vascular bundle from a donor site and anastomosing this to recipient vessels with the fixation of the donor bone to recipient bone.

### Aims and objectives

The aim of this systematic review is to examine the published evidence for the use of free VBGs in the management of nonunions of the scaphoid, specifically looking at donor site morbidity, time to union, complications and symptomatic outcomes.

## Material and methods

A search on MEDLINE and Google Scholar from 1928 to December 2013 was performed using the following search strategy: ((scaphoid nonunion) OR scaphoid pseudarthrosis) AND bone graft.

Journals in all languages were included, and there were no limitations on the search strategy. Abstracts were screened for relevance. Articles concerning the management of scaphoid nonunions with free VBGs were included.

Exclusion criteria included studies which did not separate delayed union from nonunion of the scaphoid. Letters, editorials and review articles were excluded. The number of scaphoid nonunions, type of free graft used, duration of immobilisation, type of fixation, operative technique, duration till union and postoperative complication rate were extracted from each article and compiled into a database. The references of selected full text articles were pursued for the inclusion of further articles.

Recorded data was extracted and entered into an excel spreadsheet (Microsoft Office Excel, 2007) and Graphpad statistical software (Prism 6). Statistical analysis was performed with determination of descriptive statistics, and differences between the groups were calculated using categorical variables and chi-square test. A *p* value of 0.05 or less was considered to be statistically significant.

## Results

### Selection of studies

**Figure 1:**
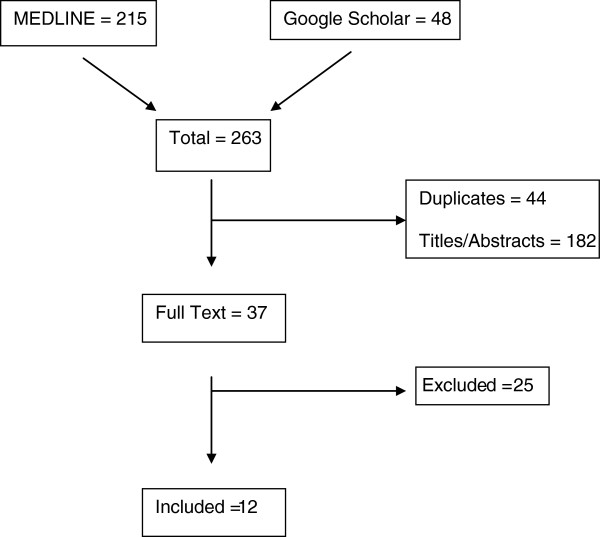
Flowchart displaying selection process.

Surgical outcomes of scaphoid nonunion only were reviewed in selected studies. The search strategy identified 263 articles. Forty-four were removed manually as duplicates. Thirty-seven full text articles were sought. Case series, case reports and prospective and retrospective studies were included. A further 25 articles were excluded as review articles or articles in which free grafts were not described. A total of 12 articles were included. Two hundred and forty-five cases of scaphoid nonunion were identified through the articles included in this systematic review (Figure 1, Table 1).

**Table 1:** **Summary of all articles included in this systematic review (*N*** **= 12)**

**Reference**	**Study type**	**Number of patients = 245**	**Technique**	**Union rate (%)**	**Average time to union (weeks)**	**Complications**	**Average follow-up (years)**	**Immobilisation (weeks)**	**Comments**
Pechlaner et al. [25]	Retrospective	25	Resection of the pseudarthrosis or the necrotic bone. Insertion and Kirschner wire fixation of corticocancellous graft from the iliac crest isolated on its vascular pedicle and anastomosed to the radial artery	25 (100%)	-	-	-	8	AVN proximal pole preoperatively
Fernandez et al. [26]	Retrospective	11	Resection of the necrotic bone and inserting a corticocancellous bone graft from the iliac crest; the second dorsal intermetacarpal artery is implanted into the proximal fragment of the scaphoid	10 (91%)	10	Nonunion and cystic area increased in 1 patient	5 years	10	Six patients had had previous unsuccessful operative attempts to obtain union
									Eight nonunions were in the proximal one third, and three at the waist of the scaphoid
Gabl et al. [23]	Retrospective	15	Microvascular pedicled iliac crest bone graft anastomosed to the radial artery after debridement of nonunion	12 (80%)	-	20% patients who failed to unite progressed to carpal collapse	6 years 1 month	12	Patients undergoing union had an average grip strength of 107 kg-force versus 73 kg-force in patients failing to unite
Gabl et al. [24]	Retrospective	56	Microvascular pedicled iliac crest bone graft anastomosed to the radial artery after debridement of nonunion	47 (85%)	-	15% patients failed to unite and progressed to carpal collapse. Arthrosis developed in all these patients	8 years 9 months follow-up	12	In those undergoing union, grip strength was 95% and range of motion 75% compared to the noninvolved wrist. Carpal collapse did not occur. Arthrosis was reduced in 25%
Harpf et al. [22]	Retrospective	60	Microvascular pedicled iliac crest bone graft anastomosed to the radial artery after debridement of nonunion. Kirschner wire fixation	55 (92%)	-	8.3% who failed to unite progressed to carpal collapse	7 years 5 months	12	61.37% of patients had bone deformations detected radiologically at the donor site, and 31.7% of patients had impairment of the lateral cutaneous nerve of the thigh
Doi et al. [21]	Retrospective	10	Free vascularised periosteal bone graft harvested from the supracondylar region of the femur and nourished by the articular branch of the descending genicular artery and vein. Kirschner wire fixation	10 (100%)	12	1 patient suffered transient knee joint stiffness, 1 patient suffered transient saphenous nerve dysaesthesias and 2 patients developed ectopic bone formation requiring resection	3 years 6 months	6	All patients had preoperative AVN proximal pole
									Mayo wrist scoring system used to score outcome and showed 8 patients with excellent or good outcomes. Grip strength returned to 33 kg-force on the affected side versus 40 kg-force on the unaffected. All patients resumed activities
Doi et al. [29]	Retrospective	11	Free vascularised periosteal bone graft consisting of periosteum, cortex and underlying cancellous bone harvested from the supracondylar region of the femur and nourished by the articular branch of the descending genicular artery and vein. Kirschner wire fixation	11 (100%)	-	-	-	Until bony union achieved	Recommends use of iliac crest donor site if large bone graft required or injury to donor site
Lanzetta [28]	Case report	1	Osteochondral-free vascularised graft from the rib based on the inferior and superior intercostal arteries. Kirschner wire fixation	1 (100%)	4	-	5 years	4	No donor site morbidity. Bone formation occurred at the radioscaphoid joint from the graft requiring radiocarpal arthrolysis. Grip strength improved by 30%
Jones et al. [30]	Retrospective	12	Free vascularised bone graft harvested from the medial supracondylar region of the femur and nourished by the articular branch of the descending genicular artery and vein. Scaphoid screw or Kirschner wire fixation	12 (100%)	13	Ectopic bone formation noted	1 year	Until bony union achieved	Comparative study against pedicled VBGs showing a significantly short time to union in free VBGs (*p* < 0.001) and a significantly higher rate of union (*p* < 0.005)
Larson et al. [31]	Retrospective	11	Free vascularised bone graft harvested from the medial supracondylar region of the femur and nourished by the articular branch of the descending genicular artery and vein. Scaphoid screw or Kirschner wire fixation	11 (100%)	12	Ectopic bone formation at periosteal flap in 2 patients	-	Until bony union achieved	Final 12th patient only 2 months postsurgery and so excluded from results
									Mayo wrist scoring system showed excellent or good results in 8 patients. No donor site morbidity
Arora et al. [27]	Retrospective	21	Microvascular pedicled iliac crest bone graft anastomosed to the radial artery after debridement of nonunion. Kirschner wire fixation	16 (76%)	19	-	5 years 7 months	12	Difference in pre- and postoperative grip strength statistically significant in patients undergoing union (*p* < 0.03). 11 of 16 patients with union showed no signs of osteoarthritis, and 5 patients showed stage 1 signs of osteoarthritis. All patients with persistent nonunion showed signs of osteoarthritis
Jones et al. [32]	Retrospective	12	Free vascularised bone graft harvested from the medial supracondylar region of the femur and nourished by the articular branch of the descending genicular artery and vein. Scaphoid screw or Kirschner wire fixation	12 (100%)	13	1 concurrent donor-site stitch abscess debridement	-	Until bony union achieved	Radial styloidectomy required after union in 1 patient

In six retrospective studies [22-27], a total of 188 patients were identified as undergoing resection of the pseudarthrosis or the necrotic bone, insertion and Kirschner wire fixation of corticocancellous graft from the iliac crest isolated on its vascular pedicle and anastomosed to the radial artery. The average union rate was 87.3% [22-27]. The largest study in these patients by Harpf et al. demonstrated that 61.37% of patients had bone deformations detected radiologically at the donor site and 31.7% of patients had impairment of the lateral cutaneous nerve of the thigh following this procedure [22]. Gabl et al. published two studies using this technique and were able to demonstrate an average grip strength of 107 kg-force in patients uniting versus 73 kg-force in patients failing to unite [23,24]. Gabl et al. also noted that range of motion was 75% compared to the noninvolved wrist in these studies and that carpal collapse did not occur [23,24].

One patient underwent an osteochondral free vascularised graft from the rib based on the inferior and superior intercostal arteries. Grip strength improved by 30% with this technique; however, radiocarpal arthrolysis was required due to ectopic bone formation. This case report had the shortest reported time of all published articles reviewed achieving union at 4 weeks [28].

In five retrospective studies [21,29-32], 56 patients underwent free vascularised periosteal bone graft consisting of periosteum, cortex, and underlying cancellous bone harvested from the supracondylar region of the femur and nourished by the articular branch of the descending geniculate artery and vein. Graft was fixed with either Kirschner wires or a scaphoid screw. There was a 100% union rate with this technique with a mean time to union of 12.5 weeks in all the studies using this technique [21,29-32]. Five out of fifty-six patients developed ectopic bone formation; however, only three patients required resection or a radial styloidectomy (*n* = 1) [21,31,32]. The overall mean union rate for all patients undergoing a free VBG in this systematic review was 93.65%. In two independent series by Doi and Larson, 8/10 patients and 8/11 patients, respectively, showed good and excellent Mayo wrist scores [21,31]. Therefore, where Mayo wrist scores were used in medial femoral condyle grafts, 76.2% of patients would have a good or excellent score.

Comparison between patients undergoing an iliac crest free VBG and patients undergoing a medial femoral VBG showed a significantly higher union rate in the medial femoral group (*p* = 0.006) (see Table 2).

**Table 2:** Number of patients treated using free VBG from iliac crest or medial femoral graft and their union rates

	**Iliac crest free VBG**	**Medial femoral free VBG**
Union	165	56
Nonunion	23	0
Total	188	56
% union	87.7	100

Chi-square = 7.564; *p* = 0.006.

## Discussion

In 1932, Gaythorne Girdlestone stated

A bone is a plant with its roots in the soft tissues and when its vascular connections are damaged it requires not the artifice of the cabinet-maker but the care and attention of the gardener [33].

Never has this poignant statement been so clinically applicable than in the use of free VBGs for nonunions. To date, various approaches have been adopted to treat scaphoid nonunions including NVBGs, dorsal and volar pedicled VBGs and free VBGs. Limitations have been encountered using the pedicled VBGs including limited degrees of manipulation and limited success in correcting humpback deformities and proximal pole AVN using more conventional techniques. Obtaining a graft of adequate size and shaping this graft to fill a defect whilst it is attached to its pedicle and correct the presence of deformities are challenging with pedicled 1,2 ICSRA VBGs. The 1,2 ICSRA pedicled VBG has yielded a range of results in the literature; however, Chang et al. provided a critical evaluation of its use, noting a 71% union rate in patients with scaphoid nonunion and 50% union in the presence of AVN [20]. Straw et al. also showed only 2 of 16 patients with AVN of the proximal pole uniting with the 1,2 ICSRA pedicled VBG [34]. The studies in Table 1 demonstrate improved results in addressing humpback deformities and proximal pole AVN with the use of free VBGs especially with the medial supracondylar free VBG.

We have noted that the union rate in medial femoral condyle VBGs is a surprising 100% in 56 patients. The higher union rate in patients undergoing medial femoral free VBGs and iliac crest free VBGs is statistically significant (*p* value = 0.006, see Table 2). This has significant implications for the challenging group of patients with proximal pole AVN as this technique so far has been successful in all the retrospective studies identified with proximal pole AVN making it an attractive management option. An understanding of the bone microcirculation is crucial to successfully use this graft. A small layer of outer cortical bone was retained in the studies as it is generally believed that this will preserve the cambium and lead to a better union rate. The medial condylar and supracondylar regions of the femur primarily depend on a medullary blood supply. Although the periosteal vessels are not the dominant source of cortical blood flow, there are anastomoses between them and the cortical vessels, and in situations of stress, a collateral circulation through periosteal vessels can be established which is sufficient to support bony union and osteocyte viability [35]. Bergrenn et al. were able to further show total bone graft survival in poorly vascularised tissues using this collateral system predominantly [29,35]. The articular branch of the descending genicular vessels and the superomedial genicular vessels can be used to base the graft upon; however, in the studies used, the descending genicular vessels were favoured due to their longer nature and their wider calibres. Doi et al. described this graft as being thin, elastic and very readily able to conform to a recipient defect [28,29]. It is likely that all these properties are responsible for the early success this graft has enjoyed in terms of the rate of union, correction of humpback deformity and functional recovery. This valuable graft has also been applied to persistent nonunions of the mandible, clavicle, humerus, ulna, metacarpals and the tibia [36-38]. However, we appreciate that these are retrospective studies with a number of uncontrolled parameters, making comparisons difficult and the potential for bias significant. Prospective studies or a randomised controlled trial comparing free VBGs is required. It is important to note that harvesting the medial femoral graft will lead to longer operative times in terms of the exposure and anastomosis; however, if two surgeons are operating simultaneously, then this may not be the case [31]. Gray et al. previously recommended the use of the free supracondylar/condylar VBG primarily in waist or distal pole nonunions in cases with AVN and with or without a humpback deformity. Given the success of medial femoral supracondylar/condylar free VBGs in proximal pole nonunions with AVN and with humpback deformities, the published data to date indicates that this graft is an appropriate option for these challenging cases [21,29-32]. Despite the disadvantages of operating on multiple sites and the need for general anaesthesia, our preference is the medial femoral condylar/supracondylar free VBG for such cases given the union rates and the mean time to union in all studies (12.5 weeks). Additionally, Jones et al. have previously reported that this graft united within 13 weeks vs 19 weeks for pedicled VBGs and that these pedicled VBGs united only 40% of the time in the presence of proximal pole AVN.

Other techniques using the iliac crest or rib as a source have also been described. However, there is only one case in the literature of a rib (including cartilage) being transplanted, and although this case report reported symptomatic relief and a good functional recovery, more cases are required before a definitive conclusion can be made regarding this osteochondral graft and any potential donor site morbidity. Also, data regarding time to union was missing from a minority of studies in the systematic review, making comparison between iliac crest grafts and the femoral grafts difficult [22,24]. In terms of assessing time to union, different surgeons adopt different times before performing an X-ray or computed tomogram (CT) scan. It is therefore difficult to say exactly when union is achieved in all grafts due to the lack of continuous monitoring. We would recommend a more routine assessment of patients with an initial X-ray at 4 to 6 weeks followed by CT scanning beyond that to detect union when suspected clinically and radiographically. Donor site pain was universal to all studies, and the use of a knee brace for 2 weeks was adopted for femoral VBGs. Five patients (8.9%) suffered ectopic bone formation with three requiring resection. However, no major complications such as fracture at the knee or instability at the wrist or knee have been reported with medial femoral VBGs [29]. The harvesting of free VBGs from the iliac crest has also yielded promising results with a mean union rate of 87.3% in 188 patients included in our study [22-27]. However, the complication rate was significantly higher in comparison to the medial femoral VBG with the largest case series of 60 patients publishing a 61.37% donor site bone deformation incidence and a 31.7% incidence of impairment of the lateral cutaneous nerve of the thigh [22]. A further limitation worth noting is that although functional outcomes have been very positive between both grafts, a minority of studies used the DASH scoring system as opposed to the Mayo system which was used in the majority of studies, making comparison difficult [21,31]. We also found that assessing graft patency postoperatively was different between centres, with some studies using a skin paddle and others using angiography or Doppler probes. It is likely that as our experience with these grafts increases, techniques to assess graft patency will also increase, but for now, a sound clinical assessment and the use of a Doppler probe should be sufficient, and where there is concern, an angiography can be performed. Both the iliac crest and medial femoral VBGs may require secondary procedures in the presence of ectopic ossification though this is not common.

## Conclusions

The medial femoral condylar/supracondylar VBG has very promising early results in terms of union rates, time to union and functional outcomes. The data suggests that this surgical option can be considered in cases of proximal pole AVN and humpback deformity or in situations where other flaps are precluded or deemed unlikely to cause union. It has wider surgical applications and has not been associated with major complications yet. A randomised controlled trial comparing the different free VBGs is desirable as this is not currently present in the literature.

## Abbreviations

AVN: Avascular necrosis; CT: Computed tomogram; NVBG: Nonvascularised bone graft; VBG: Vascularised bone graft; 1,2-ICSRA: 1,2-intercompartmental supraretinacular artery.

## Competing interests

The authors declare that they do not have any competing interests.

## Authors’ contributions

TAJ and PVG devised the project. TAJ wrote the manuscript. AM performed the statistical analysis. PVG and AM edited the manuscript. All authors read and approved the final manuscript.
